# Cognitive Reappraisal Moderates the Link Between Burden and Loneliness in Dementia Family Caregivers

**DOI:** 10.1093/geroni/igaf122.2777

**Published:** 2025-12-31

**Authors:** Ahria Dominguez, Darius Levan, Kuan-Hua Chen, Casey Brown, Jenna Wells, Julian Scheffer, Jennifer Merrilees, Robert Levenson

**Affiliations:** University of Nebraska Medical Center, Omaha, Nebraska, United States; University of California, Berkeley, Berkeley, California, United States; University of Nebraska Medical Center, Omaha, Nebraska, United States; Georgetown University, Washington, District of Columbia, United States; Cornell University, Ithaca, New York, United States; University of Western Ontario, London, Ontario, Canada; University of California, San Francisco, San Francisco, California, United States; University of California, Berkeley, Berkeley, California, United States

## Abstract

Caregiving for a loved one with a neurodegenerative disease is an important part of family life. However, the associated burden of experiencing declining functionality in a parent, spouse/partner, or other family member can lead to increased loneliness in caregivers. Cognitive reappraisal is the process of reevaluating one’s thoughts about a situation to alter their emotional experience (e.g., finding the good in a bad situation). Using cognitive reappraisal may be a protective strategy to reduce loneliness in caregivers in the context of increased burden. We studied a total of 345 caregivers (age: M = 64.44, SD = 11.46) caring for a family member with a neurodegenerative disease (parent = 90; spouse/partner = 255) who were participating in our research on dementia caregivers. Caregivers’ burden, loneliness, and likelihood of using cognitive reappraisal were assessed using well-established questionnaires. We found that greater caregiver burden was associated with greater loneliness (r = 0.436, p < 0.001). This association was moderated by caregivers’ use of cognitive reappraisal (B = -0.08, p = 0.02) such that the association between burden and loneliness was less pronounced for caregivers who were more likely to use cognitive reappraisal. This moderation was maintained when controlling for potentially confounding variables (age, gender, and education). We believe that cognitive reappraisal may buffer against loneliness by helping caregivers focus on the more positive aspects of the situation (e.g., areas of preserved functioning in the person with dementia or memories of pleasurable activities in the past).

